# Author Correction: Deep muscle-proteomic analysis of freeze-dried human muscle biopsies reveals fiber type-specific adaptations to exercise training

**DOI:** 10.1038/s41467-021-22015-4

**Published:** 2021-03-05

**Authors:** A. S. Deshmukh, D. E. Steenberg, M. Hostrup, J. B. Birk, J. K. Larsen, A. Santos, R. Kjøbsted, J. R. Hingst, C. C. Schéele, M. Murgia, B. Kiens, E. A. Richter, M. Mann, J. F. P. Wojtaszewski

**Affiliations:** 1 The Novo Nordisk Foundation Center for Protein Research, Clinical Proteomics, Faculty of Health and Medical Sciences, University of Copenhagen, Copenhagen, Denmark; 2 The Novo Nordisk Foundation Center for Basic Metablic Research, Faculty of Health and Medical Sciences, University of Copenhagen, Copenhagen, Denmark; 3 Section of Molecular Physiology, Department of Nutrition, Exercise and Sports, University of Copenhagen, Copenhagen, Denmark; 4 Section of Integrative Physiology, Department of Nutrition, Exercise and Sports, University of Copenhagen, Copenhagen, Denmark; 5 The Centre of Inflammation and Metabolism and Centre for Physical Activity Research Rigshospitalet, University Hospital of Copenhagen, Copenhagen, Denmark; 6 Department of Proteomics and Signal Transduction, Max-Planck-Institute of Biochemistry, Martinsried, Germany; 7 Department of Biomedical Sciences, University of Padua, Padua, Italy

**Keywords:** Proteomic analysis, Computational biology and bioinformatics, Skeletal muscle

Correction to: *Nature Communications* 10.1038/s41467-020-20556-8, published online 12 January 2021.

The originally published version of this Article contained an error in Figure 5. In panel 5g, a statistical significance line (indicating *P* < 0.001) was mistakenly positioned over the blue bars (Fast Pre, Fast Post), whereas it should have been positioned over the closed red and closed blue bars (Slow Post, Fast Post). The data were indicated correctly in the main text. This error has now been corrected in the PDF and HTML versions of the Article.

