# Supplementary material for: Deep muscle-proteomic analysis of freeze-dried human muscle biopsies reveals fiber type-specific adaptations to exercise training
Source: Nat Commun. 2021 Jan 12;12:304. doi: 10.1038/s41467-020-20556-8 (PMC7803955; doi:10.1038/s41467-020-20556-8)
Supplement: Supplementary file 1 — Supplementary Information [file 41467_2020_20556_MOESM1_ESM.pdf]

Deep muscle-proteomic analysis of freeze-dried human muscle biopsies reveals fiber type-specific adaptations to exercise training

Deshmukh AS[1,2$*], Steenberg DE[3$], Hostrup M[4], Birk JB[3], Larsen JK[2], Santos A[1], Kjøbsted R[3], Hingst JR[3], Schéele CC[2,5], Murgia M[6,7], Kiens B[3], Richter EA[3], Mann M[1, 6] and Wojtaszewski JFP[3*]

Supplemental Material

Supplemental Table S1 Subject characteristics describing clinical parameters.

Subjects	Age	Sex	Weight	Fasting glucose	Fasting insulin (uIU/ml)	HR rest	HR max	Total Body Fat (Kg)	% Fat	Total lean mass (Kg)
**A**	21	M	78.7	4.7	3.5	67	191	12.9	16.3	63.1
**B**	22	M	81.6	5.3	4.3	54	196	21.2	25.8	57.5
**C**	25	M	74.8	5.2	7.1	59	201	21.8	29.3	49.9
**D**	26	M	86.7	5.0	4.0	57	200	16.3	18.7	67.5
**E**	27	M	83.1	5.4	6.0	65	195	20.7	24.9	59.2

Figure S1

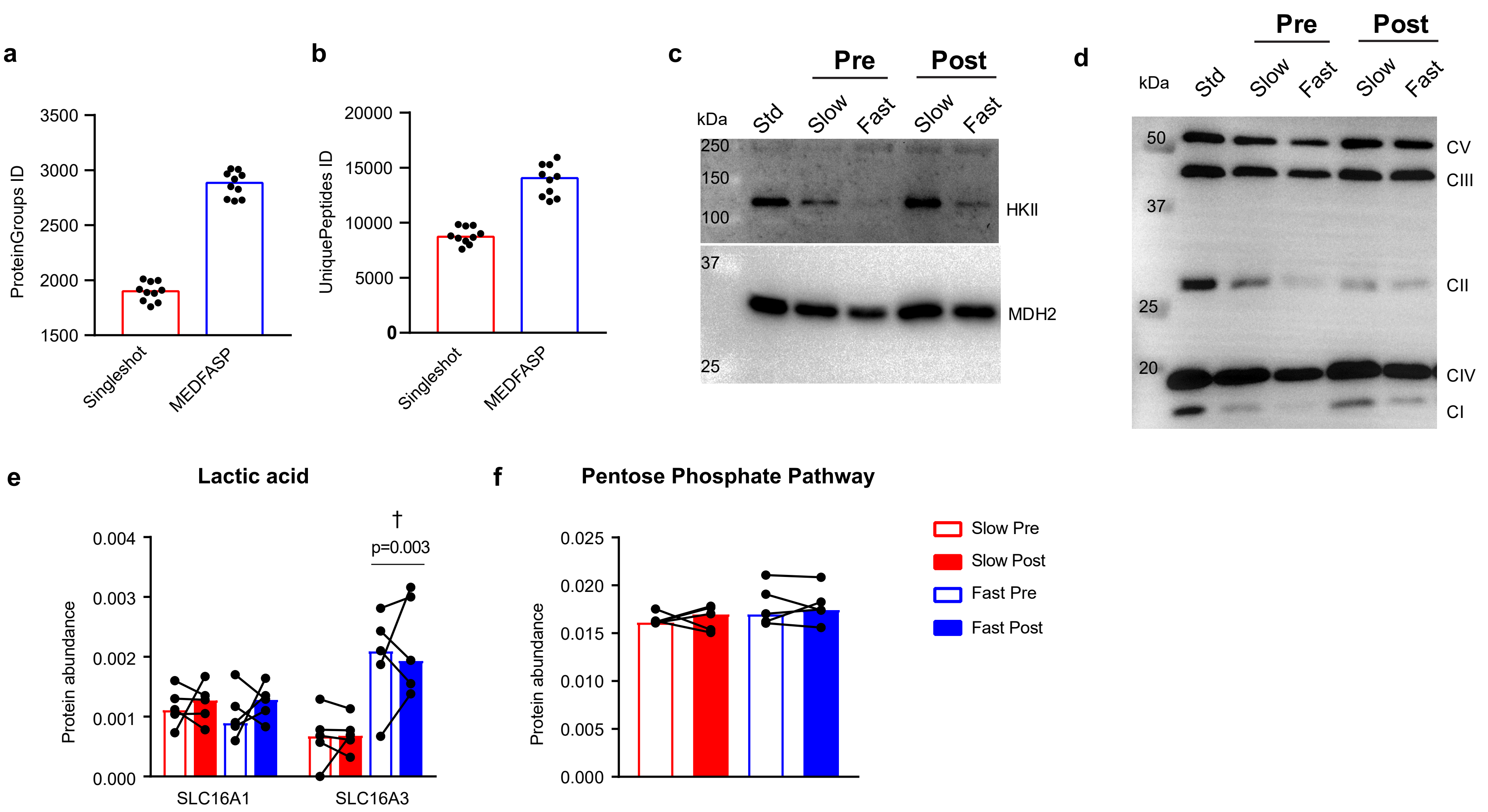

Supplementary Fig. 1 Sample preparation comparison, representative western blots and protein abundance for lactic acid metabolism and pentose phosphate pathway. Comparison of protein and unique peptide identification using single shot and MEDFASP (multi-enzyme digestion filter-aided sample preparation) protocol analyzed from total muscle lysate (n=10 (5 PRE + 5 POST)) (a-b). Representative Western blots for HKII, MDH2 (c) and OXPHOS subunits CI-CV (d) in slow and fast muscle fibers under PRE and Post conditions (c-d-). Normalized protein abundance of enzymes involved in lactic acid metabolism (n=5,e) and pentose phosphate pathways (n=5, f). Data in figures are shown as median. P-values by two-way RM ANOVA are indicated in figure S1E. "*" denotes a training effectLines indicate main effects.

Figure S2

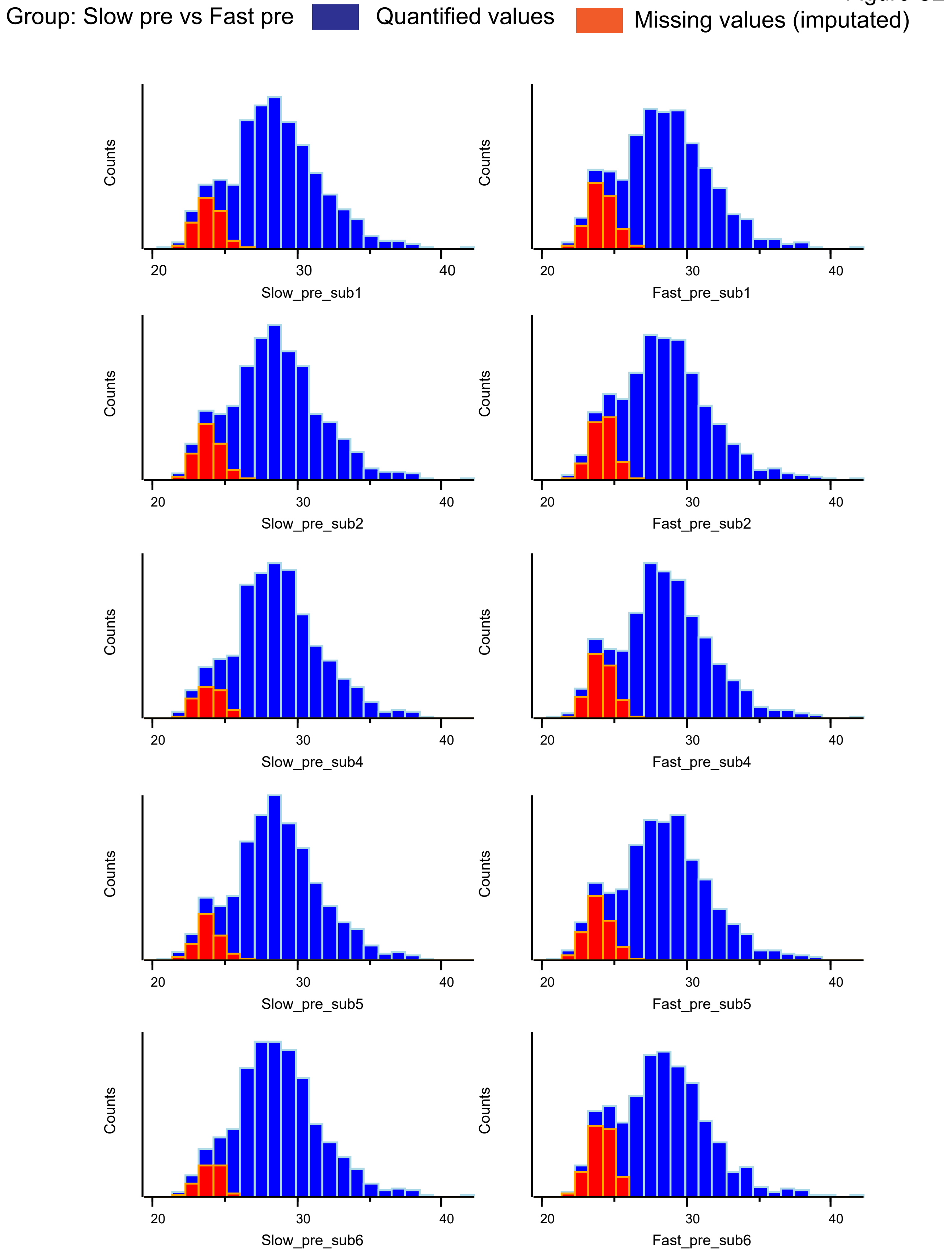

Supplementary Fig. 2 Distribution of imputed and non imputed data for slow and fast fibers under PRE condition. Histogram representing quantified (in blue) and imputed (in orange) values in slow PRE and fast PRE groups (n=5). Each histogram corresponds to one subject, one fiber type (Slow/Fast) and one biological condition (PRE).

Figure S3

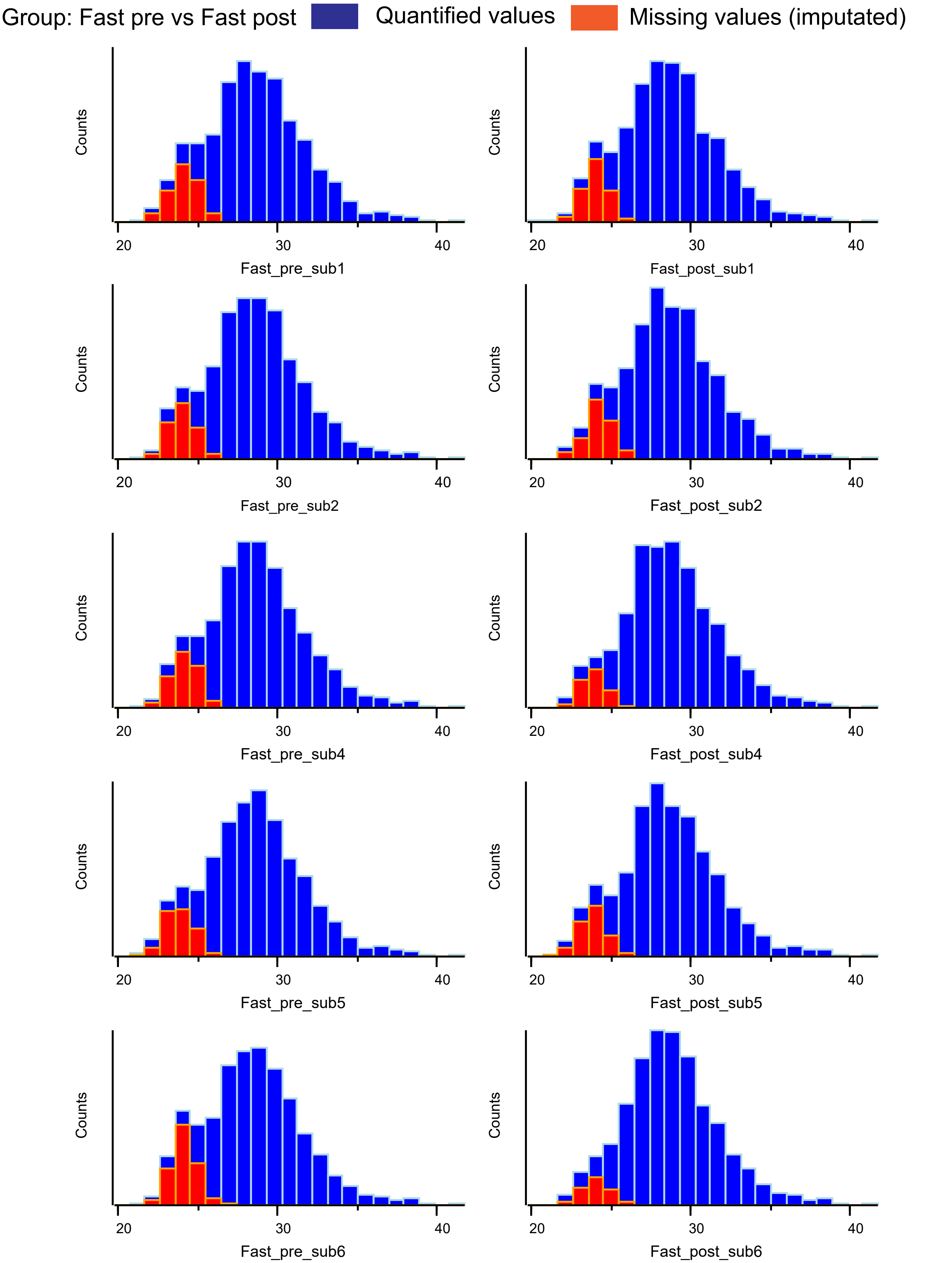

Supplementary Fig. 3 Distribution of imputed and non imputed data for fast fibers under PRE and POST condition. Histogram representing quantified (in blue) and imputed (in orange) values in fast PRE and fast POST group (n=5). Each histogram corresponds to one subject, fast fiber type (Slow/Fast) and one biological condition (PRE/POST).

Figure S4

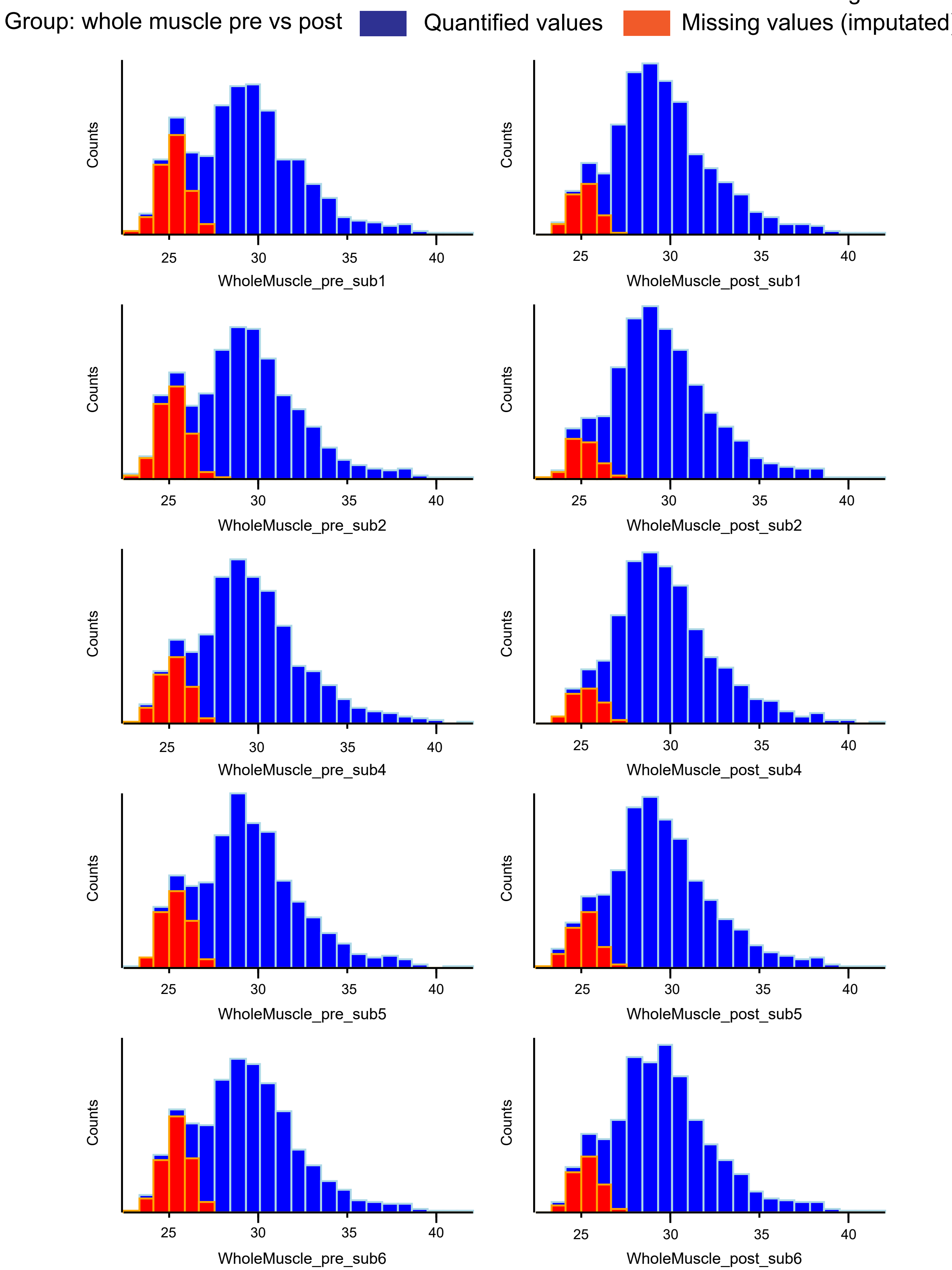

Supplementary Fig. 4 Distribution of imputed and non imputed data for whole muscle under PRE and POST condition. Histogram representing quantified (in blue) and imputed (in orange) values in whole muscle PRE and POST group (n=5). Each histogram corresponds to one subject, and one biological condition (PRE/POST).
