# Supplementary material for: Deep muscle-proteomic analysis of freeze-dried human muscle biopsies reveals fiber type-specific adaptations to exercise training
Source: Nat Commun. 2021 Jan 12;12:304. doi: 10.1038/s41467-020-20556-8 (PMC7803955; doi:10.1038/s41467-020-20556-8)
Supplement: Supplementary file 3 — Description of Additional Supplementary Files [file 41467_2020_20556_MOESM3_ESM.pdf]

Title: Supplemental Data 1
Description: The list of total proteins identified in muscle fibers and human primary muscle cells.

Title: Supplemental Data 2
Description: Comparison of protein identification by singleshot and MEDFASP protocol in whole muscle lysate

Title: Supplemental Data 3
Description: Cumulative protein abundance of all proteins in slow and fast muscle fibers.

Title: Supplemental Data 4
Description: Pearson correlation (r) between the replicates

Title: Supplemental Data 5
Description: The list of significantly different proteins between slow and fast muscle fibers (paired t test) + Enriched categories after Fischer Exact Test + non-imputated LFQ values for quantified proteins

Title: Supplemental Data 6
Description: Members of individual organelle/comparement presented in figure 2

Title: Supplemental Data 7
Description: The list of significantly different proteins in slow fibers, fast fibers and whole muscle lysate after exercise (paired t test) + non imputated matrices displaying protein quantification for slow fiber, fast fiber and whole muscle lysate.

Title: Supplemental Data 8
Description: Output file of ClueGo enrichment analysis

Title: Supplemental Data 9
Description: The list of the proteins adapated to exercise training in fiber type specific manner + list of the proteins which were exclusively quantified in either slow or fast muscle fiber + log2 fold change for significantly different proteins

Title: Supplemental Data 10
Description: Members of individual protein categories presented in figure 5

Title: Supplemental Data 11
Description: Fold change, significance score for enzymes from glycolysis, PHD complex and TCA cycle in slow and fast muscle fibers.
