# Supplementary material for: Deep muscle-proteomic analysis of freeze-dried human muscle biopsies reveals fiber type-specific adaptations to exercise training
Source: Nat Commun. 2021 Jan 12;12:304. doi: 10.1038/s41467-020-20556-8 (PMC7803955; doi:10.1038/s41467-020-20556-8)
Supplement: Supplementary file 15 — Reporting Summary [file 41467_2020_20556_MOESM15_ESM.pdf]

nature research

Corresponding author(s): Atul S Deshmukh
Jørgen Wojtaszewski

Last updated by author(s): Nov 8, 2020

Reporting Summary

Nature Research wishes to improve the reproducibility of the work that we publish. This form provides structure for consistency and transparency in reporting. For further information on Nature Research policies, see Authors & Referees and the Editorial Policy Checklist .

Statistics

For all statistical analyses, confirm that the following items are present in the figure legend, table legend, main text, or Methods section.

n/a	Confirmed	
☐	☒	The exact sample size (*n*) for each experimental group/condition, given as a discrete number and unit of measurement
☐	☒	A statement on whether measurements were taken from distinct samples or whether the same sample was measured repeatedly
☐	☒	The statistical test(s) used AND whether they are one- or two-sided<br>*Only common tests should be described solely by name; describe more complex techniques in the Methods section.*
☒	☐	A description of all covariates tested
☐	☒	A description of any assumptions or corrections, such as tests of normality and adjustment for multiple comparisons
☐	☒	A full description of the statistical parameters including central tendency (e.g. means) or other basic estimates (e.g. regression coefficient) AND variation (e.g. standard deviation) or associated estimates of uncertainty (e.g. confidence intervals)
☐	☒	For null hypothesis testing, the test statistic (e.g. *F*, *t*, *r*) with confidence intervals, effect sizes, degrees of freedom and *P* value noted<br>*Give P values as exact values whenever suitable.*
☒	☐	For Bayesian analysis, information on the choice of priors and Markov chain Monte Carlo settings
☒	☐	For hierarchical and complex designs, identification of the appropriate level for tests and full reporting of outcomes
☐	☒	Estimates of effect sizes (e.g. Cohen's *d*, Pearson's *r*), indicating how they were calculated

Our web collection on statistics for biologists contains articles on many of the points above.

Software and code

Policy information about availability of computer code

Data collection	MaxQuant (https://maxquant.org/) version - 1.5.2.8
Data analysis	Perseus (http://www.coxdocs.org/doku.php?id=perseus:start) Version 1.6.0.8, Cytoscape 3.6.1, Graphpad Prism 7, Adobe illustrator CS6

For manuscripts utilizing custom algorithms or software that are central to the research but not yet described in published literature, software must be made available to editors/reviewers. We strongly encourage code deposition in a community repository (e.g. GitHub). See the Nature Research guidelines for submitting code & software for further information.

Data

Policy information about availability of data

All manuscripts must include a data availability statement. This statement should provide the following information, where applicable:

- Accession codes, unique identifiers, or web links for publicly available datasets
- A list of figures that have associated raw data
- A description of any restrictions on data availability

The mass spectrometry proteomics data have been deposited at the ProteomeXchange Consortium (http://proteomecentral.proteomexchange.org) via the PRIDE partner repository with the dataset identifier PXD012824, Username: reviewer39199@ebi.ac.uk, Password: W8amRKi4. All figures in the manuscript are associated with this data.Tandem mass spectra were searched against the Uniprot human databases (Version June 2015)

Field-specific reporting

Please select the one below that is the best fit for your research. If you are not sure, read the appropriate sections before making your selection.

Life sciences study design

All studies must disclose on these points even when the disclosure is negative.

Sample size	We analyzed muscle samples from five healthy males. No samples calculation was performed. Muscle samples were analyzed before and after exercise training. Since we analyzed LysC and Trypsin digested peptides from slow and fast muscle fibers (before and after digestion), we selected n-5. Given restraints related to instrumentation time, resources, and time to undertake single fiber isolation and subsequent mass-spectrometry as well as limited biological material, five subjects from the original study were selected for the present analysis. In our experience, for this protocol, this n size is sufficient to provide detailed proteome adaptation in skeletal muscle (Schönke et al Proteomics 2018, Gonazalez-Franquesa et al bioRxiv (https://doi.org/10.1101/860080)
Data exclusions	No data was excluded.
Replication	Due to difficulties in isolating and typifying single muscle fibers, limited availability of the biopsies material, and limitations on the LCMS instrumentation time, all experiments were performed only once for five subjects under PRE and POST conditions. Nevertheless, we verified the reproducibility of the proteomics data within biological replicates using Pearson correlation analysis, principal component analysis as well the number of peptide and protein identification for each replicates. All attempt used for checking the reproducibility in this regards were successful.
Randomization	Randomization was applied for the study.
Blinding	Yes, the investigators were blinded to the group.

Reporting for specific materials, systems and methods

We require information from authors about some types of materials, experimental systems and methods used in many studies. Here, indicate whether each material, system or method listed is relevant to your study. If you are not sure if a list item applies to your research, read the appropriate section before selecting a response.

Materials & experimental systems

n/a	Involved in the study	
☐	☒	Antibodies
☒	☐	Eukaryotic cell lines
☒	☐	Palaeontology
☒	☐	Animals and other organisms
☐	☒	Human research participants
☒	☐	Clinical data

Methods

n/a	Involved in the study	
☒	☐	ChIP-seq
☒	☐	Flow cytometry
☒	☐	MRI-based neuroimaging

Antibodies

Antibodies used

Monoclonal mouse anti-myosin heavy chain (human slow fibers), Developmental Studies Hybridoma Bank, Cat.# A4.840-c, Lot.# 10/21/10, RRID AB_528384
Monoclonal mouse anti-myosin heavy chain (human fast fibers), Developmental Studies Hybridoma Bank, Cat.# A4.74-c, Lot.# 10/29/10, RRID AB_528383.
Monoclonal mouse anti-glycogen, Kindly, provided by Dr. Otto Baba, Tokyo Medical and Dental University, Tokyo, Japan.
Total OXPHOS Human WB Antibody Cocktail, Abcam Cat.# ab110411, Lot.# J5383, RRID AB_2756818.
Hexokinase II, Cell Signaling Technology, Cat.# 2867, Clone C64G5, Lot.# 3, RRID AB_2232946.
MDH2, Abcam, Cat.# ab181873, Clone EPR14882(B), Lot.# n/a, RRID AB
Goat-Anti-Mouse-HRP, Jackson ImmunoResearch Labs, Cat# 111-035-062, Lot# 124708, RRIDAB_2338504
Goat-Anti-Rabbit-HRP, Jackson ImmunoResearch Labs, Cat# 111-035-045, Lot# 129786, RRIDAB_2337938
Donkey-Anti-Sheep-HRP, Jackson ImmunoResearch Labs, Cat# 713-035-147, Lot# 125113, RRID AB_2340710
Rabbit-Anti-Goat, Agilent, Cat# P0449, RRID AB_2617143
Rabit anti-mouse IgG biotinylated,DAKO, Cat# E0354, Lot# 70978, RRID AB_2687571
Goat anti-mouse IgM biotinylated, Jackson ImmunoResearch Labs, Cat# 115-065-075, Lot# 94952, RRID AB_2338566
Strepavidin-HRP, Jackson ImmunoResearch Labs, Cat# 016-030-084, Lot# 94813, RRID AB_2337238

Validation

Monoclonal mouse anti-myosin heavy chain (human slow fibers), Developmental Studies Hybridoma Bank, Cat.# A4.840-c, Lot.# 10/21/10, RRID AB_528384. We have verified this antibody with band alignment between human and mouse skeletal muscle samples on stain free gels and blotted membranes with antibody (unpublished data). We have used this antibody in the following publications with expected results: doi: 10.2337/db14-0590. Epub 2014 Sep 3. PMID: 25187364, doi: 10.1113/jphysiol.2014.283267. Epub 2015 Feb 27. PMID: 25640469, doi: 10.1113/JP280475. Online ahead of print. PMID: 32916040.

Furthermore, the manufacture has 40 citations for this antibody on their website: https://dshb.biology.uiowa.edu/A4-840

Monoclonal mouse anti-myosin heavy chain (human fast fibers), Developmental Studies Hybridoma Bank, Cat.# A4.74-c, Lot.# 10/29/10, RRID AB_528383. We have verified this antibody with band alignment between human and mouse skeletal muscle samples on stain free gels and blotted membranes with antibody (unpublished data). We have used this antibody in the following publications with expected results: doi: 10.2337/db14-0590. Epub 2014 Sep 3. PMID: 25187364, doi: 10.1113/jphysiol.2014.283267. Epub 2015 Feb 27. PMID: 25640469, doi: 10.1113/JP280475. PMID: 32916040. Furthermore, the manufacture has 36 citations for this antibody on their website: https://dshb.biology.uiowa.edu/A4-74

Monoclonal mouse anti-glycogen, Kindly, provided by Dr. Otto Baba, Tokyo Medical and Dental University, Tokyo, Japan. This is a noncommercial antibody, so it has no registration ID. We have verified this antibody on dotblot with human skeletal muscle samples with known glycogen content and shown that the antibody gives corresponding results. We have used this antibody in the following publications with expected results: doi: 10.2337/db14-0590. Epub 2014 Sep 3. PMID: 25187364, doi: 10.1113/jphysiol.2014.283267. Epub 2015 Feb 27. PMID: 25640469, doi: 10.1113/JP280475. Online ahead of print. PMID: 32916040

Total OXPHOS Human WB Antibody Cocktail, Abcam Cat.# ab110411, Lot.# J5383, RRID AB_2756818. This ab cocktail consists of five very specific monoclonal antibodies directed against different subunits of the five OXPHOS complexes in human mitochondria. It has also been tested positive on isolated mitochondria from human HCT116 cells. We have used this antibody in the following publication with expected results: doi: 10.1113/JP276735. Epub 2018 Nov 22. PMID: 30325018. The manufacturer has 268 citations on their website for this product:
https://www.abcam.com/total-oxphos-human-wb-antibody-cocktail-ab110411.html?productwalltab=abreviews&productWallTab=Abreviews

Hexokinase II, Cell Signaling Technology, Cat.# 2867, Clone C64G5, Lot.# 3, RRID AB_2232946. We have used this antibody in the following publications with expected results: doi: 10.2337/db14-0590. Epub 2014 Sep 3. PMID: 25187364, doi: 10.1016/j.molmet.2018.07.001. Epub 2018 Jul 25. PMID: 30093357, doi: 10.1113/JP276735. Epub 2018 Nov 22. PMID: 30325018.Furthermore, the manufacture has 72 citations on WB for this antibody on their website: https://www.cellsignal.com/products/primary-antibodies/hexokinase-ii-c64g5-rabbit-mab/2867?Ntk=Products&Ntt=2867

MDH2, Abcam, Cat.# ab181873, Clone EPR14882(B), Lot.# n/a, RRID AB. The manufacturer has tested this antibody with WT HEK293T cells and MDH2 knockout HEK293T cells, showing that the only band (at the expected MW) is absent in the KO cells. They also show that the antibody recognizes recombinant human MDH2 expressed in E. coli and that it runs at the expected MW (35 kDa). When we blot on our human skeletal muscle samples, we only see one band on the membrane and this is at 35 kDa.The manufacture has two citations for this antibody on their website: https://www.abcam.com/mdh2-antibody-epr14882b-ab181873.html

Human research participants

Policy information about studies involving human research participants

Population characteristics	Young (24±1(mean±SEM) years), lean (BMI: 24.2±0.4 (mean±SEM) kg m–2), untrained (VO2peak: 43.5±2.5 ml/kg/min (mean ±SEM) and healthy male subjects (n=5) were studied.
Recruitment	The subjects were recruited from the Copenhagen area in Denmark. The subjects gave their written, informed consent to participate in the study approved by the Regional Ethics Committee for Copenhagen (H-6-2014-038) and complied with the ethical guidelines of the Declaration of Helsinki II. The recruitment was based on the following inclusion and exclusion criteria:<br>Inclusion criteria were:<br>• Healthy males, non-smokers<br>• 20-30 years<br>• BMI between 20-25 kg/m2<br>• Untrained/moderately trained (VO2peak between 35-50 ml/kg/min)<br>• No family history of diseases (cardiovascular diseases, diabetes or other metabolic diseases)<br>• No medication<br><br>The exclusion criteria were:<br>• Females<br>• Smokers<br>• Males younger than 20 or older than 30 years<br>• BMI below 20 or above 25 kg/m2<br>• VO2peak below 35 or above 50 ml/kg/min
Ethics oversight	Copenhagen University, Department for Nutrition, exercise and Sport.

Note that full information on the approval of the study protocol must also be provided in the manuscript.
